# 5-Fluoro-1*H*-indole-3-carb­oxy­lic acid

**DOI:** 10.1107/S1600536811053426

**Published:** 2011-12-21

**Authors:** Wen-Jun Lu, Zhi-Hong Zou, Yang-Hui Luo

**Affiliations:** a Ordered Matter Science Research Center, College of Chemistry and Chemical Engineering, Southeast University, Nanjing 210096, People’s Republic of China

## Abstract

In the title compound, C_9_H_6_FNO_2_, the carboxyl group is twisted slightly away from the indole-ring plane [dihedral angle = 7.39 (10)°]. In the crystal, carboxyl inversion dimers linked by pairs of O—H⋯O hydrogen bonds generate *R*_2_^2^(8) loops and N—H⋯O hydrogen bonds connect the dimers into (10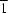) sheets.

## Related literature

For background to indoles as pharmaceuticals, see: Lang *et al.* (2011 ▶).
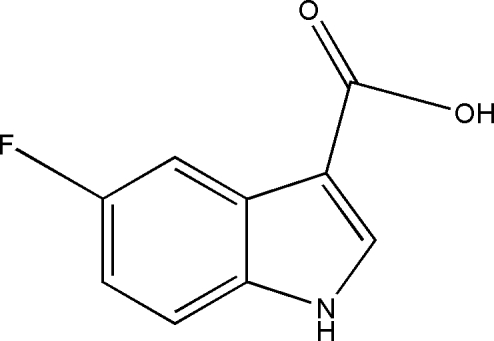


## Experimental

### 

#### Crystal data

C_9_H_6_FNO_2_
*M* *_r_* = 179.15
Monoclinic, 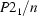

*a* = 4.4176 (9) Å
*b* = 11.073 (2) Å
*c* = 16.014 (3) Å
β = 96.63 (3)°
*V* = 778.1 (3) Å^3^
*Z* = 4
Mo *K*α radiation
μ = 0.13 mm^−1^
*T* = 293 K
0.30 × 0.20 × 0.10 mm

#### Data collection

Rigaku SCXmini CCD diffractometer
Absorption correction: multi-scan (*CrystalClear*; Rigaku, 2005 ▶) *T*_min_ = 0.982, *T*_max_ = 0.993
7874 measured reflections
1788 independent reflections
1153 reflections with *I* > 2σ(*I*)
*R*_int_ = 0.068

#### Refinement

*R*[*F* ^2^ > 2σ(*F* ^2^)] = 0.054
*wR*(*F* ^2^) = 0.137
*S* = 1.02
1788 reflections
123 parameters
H atoms treated by a mixture of independent and constrained refinement
Δρ_max_ = 0.18 e Å^−3^
Δρ_min_ = −0.26 e Å^−3^

### 

Data collection: *CrystalClear* (Rigaku, 2005 ▶); cell refinement: *CrystalClear*; data reduction: *CrystalClear*; program(s) used to solve structure: *SHELXS97* (Sheldrick, 2008 ▶); program(s) used to refine structure: *SHELXL97* (Sheldrick, 2008 ▶); molecular graphics: *DIAMOND* (Brandenburg & Putz, 2005 ▶); software used to prepare material for publication: *SHELXL97*.

## Supplementary Material

Crystal structure: contains datablock(s) I, global. DOI: 10.1107/S1600536811053426/hb6548sup1.cif

Structure factors: contains datablock(s) I. DOI: 10.1107/S1600536811053426/hb6548Isup2.hkl

Supplementary material file. DOI: 10.1107/S1600536811053426/hb6548Isup3.cml

Additional supplementary materials:  crystallographic information; 3D view; checkCIF report

## Figures and Tables

**Table 1:** Hydrogen-bond geometry (Å, °)

*D*—H⋯*A*	*D*—H	H⋯*A*	*D*⋯*A*	*D*—H⋯*A*
O1—H1⋯O2^i^	0.82	1.86	2.669 (2)	171
N1—H1*B*⋯O2^ii^	0.89 (3)	2.18 (3)	3.026 (2)	159 (2)

Symmetry codes: (i) 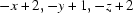; (ii) 
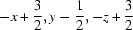
.

**Fig. 1.**
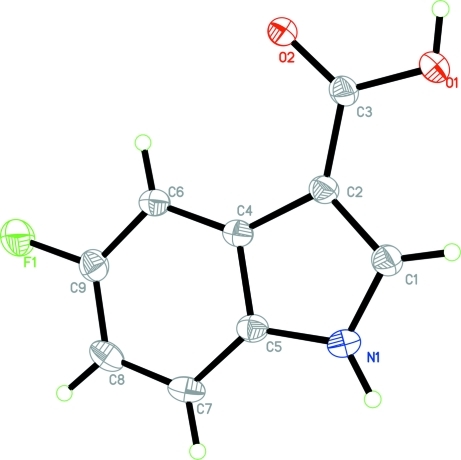
The molecular structure of the title compound with displacement ellipsoids drawn at the 30% probability level.

**Fig. 2.**
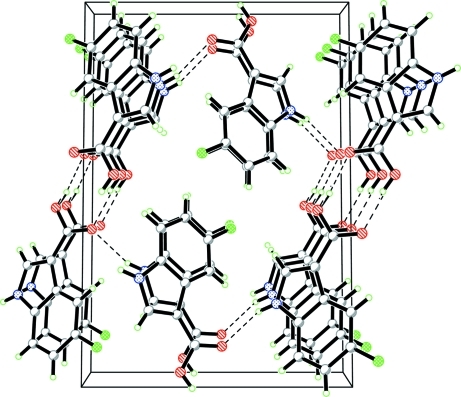
A packing view down the a axis showing hydrogen bonds as dashed lines.

## supplementary crystallographic information

### Experimental

A commercial sample of the title compound was obtained. Colourlss prisms were obtained by slow evaporation of a methanol solution over a period of seven days.

### Refinement

All H atoms attached to C atoms and O atoms were fixed geometrically and treated as riding with C—H = 0.93 Å (CH), N—H = 0.86 Å and O—H = 0.86 Å with *U*_iso_(H) = 1.2*U*_eq_(CH), *U*_iso_(H) = 1.3*U*_eq_(N) and *U*_iso_(H) = 1.5*U*_eq_(O).

### Crystal data

**Table:**

C_9_H_6_FNO_2_	*F*(000) = 368
*M**_r_* = 179.15	*D*_x_ = 1.529 Mg m^−^^3^
Monoclinic, *P*2_1_/*n*	Mo *K*α radiation, λ = 0.71073 Å
Hall symbol: -P 2yn	Cell parameters from 1954 reflections
*a* = 4.4176 (9) Å	θ = 3.2–25.0°
*b* = 11.073 (2) Å	µ = 0.13 mm^−^^1^
*c* = 16.014 (3) Å	*T* = 293 K
β = 96.63 (3)°	Prism, colourless
*V* = 778.1 (3) Å^3^	0.30 × 0.20 × 0.10 mm
*Z* = 4	

### Data collection

**Table:**

Rigaku SCXmini CCD diffractometer	1788 independent reflections
Radiation source: fine-focus sealed tube	1153 reflections with *I* > 2σ(*I*)
graphite	*R*_int_ = 0.068
Detector resolution: 13.6612 pixels mm^-1^	θ_max_ = 27.5°, θ_min_ = 3.2°
CCD profile–fitting scans	*h* = −5→5
Absorption correction: multi-scan (*CrystalClear*; Rigaku, 2005)	*k* = −14→14
*T*_min_ = 0.982, *T*_max_ = 0.993	*l* = −20→20
7874 measured reflections	

### Refinement

**Table:**

Refinement on *F*^2^	Primary atom site location: structure-invariant direct methods
Least-squares matrix: full	Secondary atom site location: difference Fourier map
*R*[*F*^2^ > 2σ(*F*^2^)] = 0.054	Hydrogen site location: inferred from neighbouring sites
*wR*(*F*^2^) = 0.137	H atoms treated by a mixture of independent and constrained refinement
*S* = 1.02	*w* = 1/[σ^2^(*F*_o_^2^) + (0.0575*P*)^2^ + 0.142*P*] where *P* = (*F*_o_^2^ + 2*F*_c_^2^)/3
1788 reflections	(Δ/σ)_max_ < 0.001
123 parameters	Δρ_max_ = 0.18 e Å^−^^3^
0 restraints	Δρ_min_ = −0.26 e Å^−^^3^

### Special details

**Table:**

Geometry. All e.s.d.'s (except the e.s.d. in the dihedral angle between two l.s. planes) are estimated using the full covariance matrix. The cell e.s.d.'s are taken into account individually in the estimation of e.s.d.'s in distances, angles and torsion angles; correlations between e.s.d.'s in cell parameters are only used when they are defined by crystal symmetry. An approximate (isotropic) treatment of cell e.s.d.'s is used for estimating e.s.d.'s involving l.s. planes.
Refinement. Refinement of *F*^2^ against ALL reflections. The weighted *R*-factor *wR* and goodness of fit *S* are based on *F*^2^, conventional *R*-factors *R* are based on *F*, with *F* set to zero for negative *F*^2^. The threshold expression of *F*^2^ > σ(*F*^2^) is used only for calculating *R*-factors(gt) *etc*. and is not relevant to the choice of reflections for refinement. *R*-factors based on *F*^2^ are statistically about twice as large as those based on *F*, and *R*- factors based on ALL data will be even larger.

### Fractional atomic coordinates and isotropic or equivalent isotropic displacement parameters (Å^2^)

**Table:**

	*x*	*y*	*z*	*U*_iso_*/*U*_eq_	
O2	1.0621 (4)	0.51786 (13)	0.89866 (9)	0.0431 (4)	
F1	1.4484 (3)	0.55381 (14)	0.59940 (9)	0.0624 (5)	
N1	0.7010 (4)	0.21726 (17)	0.71693 (12)	0.0388 (5)	
C4	1.0013 (4)	0.38262 (17)	0.73456 (12)	0.0295 (5)	
C7	0.9558 (5)	0.2922 (2)	0.59302 (14)	0.0410 (6)	
H7	0.8767	0.2328	0.5556	0.049*	
O1	0.8085 (4)	0.36747 (14)	0.95496 (9)	0.0544 (5)	
H1	0.8432	0.4097	0.9970	0.082*	
C5	0.8845 (5)	0.29428 (17)	0.67573 (14)	0.0322 (5)	
C3	0.9254 (5)	0.41935 (19)	0.89110 (13)	0.0341 (5)	
C6	1.1989 (4)	0.47175 (18)	0.70871 (13)	0.0333 (5)	
H6	1.2837	0.5308	0.7456	0.040*	
C2	0.8776 (5)	0.35426 (18)	0.81256 (13)	0.0320 (5)	
C1	0.6979 (5)	0.25319 (19)	0.79748 (14)	0.0367 (5)	
H1A	0.5906	0.2154	0.8368	0.044*	
C9	1.2604 (5)	0.4674 (2)	0.62638 (14)	0.0387 (5)	
C8	1.1466 (5)	0.3805 (2)	0.56824 (14)	0.0441 (6)	
H8	1.1984	0.3819	0.5136	0.053*	
H1B	0.599 (5)	0.154 (2)	0.6945 (16)	0.052 (7)*	

### Atomic displacement parameters (Å^2^)

**Table:**

	*U*^11^	*U*^22^	*U*^33^	*U*^12^	*U*^13^	*U*^23^
O2	0.0677 (11)	0.0344 (9)	0.0267 (8)	−0.0101 (8)	0.0033 (8)	−0.0006 (6)
F1	0.0725 (10)	0.0703 (10)	0.0478 (9)	−0.0232 (8)	0.0220 (8)	0.0004 (7)
N1	0.0464 (11)	0.0336 (10)	0.0360 (11)	−0.0070 (9)	0.0027 (9)	−0.0069 (8)
C4	0.0299 (10)	0.0297 (11)	0.0280 (11)	0.0059 (8)	−0.0011 (9)	−0.0021 (9)
C7	0.0449 (13)	0.0454 (14)	0.0320 (13)	0.0019 (11)	0.0012 (10)	−0.0135 (10)
O1	0.0903 (14)	0.0477 (10)	0.0262 (9)	−0.0222 (9)	0.0116 (9)	−0.0019 (7)
C5	0.0328 (11)	0.0304 (11)	0.0326 (12)	0.0038 (9)	0.0005 (9)	−0.0034 (9)
C3	0.0436 (12)	0.0324 (11)	0.0253 (11)	0.0029 (10)	0.0004 (9)	0.0039 (9)
C6	0.0339 (11)	0.0332 (11)	0.0318 (12)	0.0017 (9)	−0.0002 (9)	−0.0039 (9)
C2	0.0383 (11)	0.0298 (11)	0.0270 (11)	0.0016 (9)	0.0006 (9)	−0.0002 (9)
C1	0.0449 (12)	0.0331 (11)	0.0322 (12)	0.0008 (10)	0.0046 (10)	0.0006 (9)
C9	0.0378 (12)	0.0425 (13)	0.0372 (13)	−0.0015 (10)	0.0099 (10)	0.0018 (10)
C8	0.0489 (14)	0.0565 (15)	0.0281 (12)	0.0038 (12)	0.0092 (11)	−0.0048 (11)

### Geometric parameters (Å, °)

**Table:**

O2—C3	1.246 (2)	C7—H7	0.9300
F1—C9	1.369 (2)	O1—C3	1.328 (2)
N1—C1	1.351 (3)	O1—H1	0.8200
N1—C5	1.394 (3)	C3—C2	1.444 (3)
N1—H1B	0.89 (3)	C6—C9	1.378 (3)
C4—C5	1.414 (3)	C6—H6	0.9300
C4—C6	1.411 (3)	C2—C1	1.377 (3)
C4—C2	1.454 (3)	C1—H1A	0.9300
C7—C8	1.378 (3)	C9—C8	1.392 (3)
C7—C5	1.397 (3)	C8—H8	0.9300
			
C1—N1—C5	110.02 (18)	C9—C6—C4	116.95 (19)
C1—N1—H1B	123.9 (16)	C9—C6—H6	121.5
C5—N1—H1B	126.1 (16)	C4—C6—H6	121.5
C5—C4—C6	118.62 (19)	C1—C2—C3	125.57 (19)
C5—C4—C2	106.17 (18)	C1—C2—C4	106.92 (18)
C6—C4—C2	135.20 (19)	C3—C2—C4	127.49 (19)
C8—C7—C5	118.3 (2)	N1—C1—C2	109.7 (2)
C8—C7—H7	120.9	N1—C1—H1A	125.1
C5—C7—H7	120.9	C2—C1—H1A	125.1
C3—O1—H1	109.5	F1—C9—C6	118.10 (19)
C7—C5—N1	130.24 (19)	F1—C9—C8	117.23 (19)
C7—C5—C4	122.58 (19)	C6—C9—C8	124.7 (2)
N1—C5—C4	107.18 (18)	C7—C8—C9	118.9 (2)
O2—C3—O1	122.08 (19)	C7—C8—H8	120.6
O2—C3—C2	122.83 (19)	C9—C8—H8	120.6
O1—C3—C2	115.09 (19)		

### Hydrogen-bond geometry (Å, °)

**Table:**

*D*—H···*A*	*D*—H	H···*A*	*D*···*A*	*D*—H···*A*
O1—H1···O2^i^	0.82	1.86	2.669 (2)	171
N1—H1B···O2^ii^	0.89 (3)	2.18 (3)	3.026 (2)	159 (2)

Symmetry codes: (i) −*x*+2, −*y*+1, −*z*+2; (ii) −*x*+3/2, *y*−1/2, −*z*+3/2.
